# The Success of Linear Bootstrapping Models: Decision Domain-, Expertise-, and Criterion-Specific Meta-Analysis

**DOI:** 10.1371/journal.pone.0157914

**Published:** 2016-06-21

**Authors:** Esther Kaufmann, Werner W. Wittmann

**Affiliations:** 1 Institute of Education, University of Zurich, Zurich, Switzerland; 2 Otto-Selz Institute for Applied Psychology, University of Mannheim, Mannheim, Germany; Cardiff University, UNITED KINGDOM

## Abstract

The success of bootstrapping or replacing a human judge with a model (e.g., an equation) has been demonstrated in Paul Meehl’s (1954) seminal work and bolstered by the results of several meta-analyses. To date, however, analyses considering different types of meta-analyses as well as the potential dependence of bootstrapping success on the decision domain, the level of expertise of the human judge, and the criterion for what constitutes an accurate decision have been missing from the literature. In this study, we addressed these research gaps by conducting a meta-analysis of lens model studies. We compared the results of a traditional (bare-bones) meta-analysis with findings of a meta-analysis of the success of bootstrap models corrected for various methodological artifacts. In line with previous studies, we found that bootstrapping was more successful than human judgment. Furthermore, bootstrapping was more successful in studies with an objective decision criterion than in studies with subjective or test score criteria. We did not find clear evidence that the success of bootstrapping depended on the decision domain (e.g., education or medicine) or on the judge’s level of expertise (novice or expert). Correction of methodological artifacts increased the estimated success of bootstrapping, suggesting that previous analyses without artifact correction (i.e., traditional meta-analyses) may have underestimated the value of bootstrapping models.

## Introduction

Across a variety of settings, human judges are often replaced or ‘bootstrapped’ by decision-making models (e.g., equations) in order to increase the accuracy of important—and often ambiguous—decisions, such as reaching a medical diagnosis or choosing a candidate for a particular job (see [1]). Before we outline our work on the success of bootstrapping models, it should be noted that the term bootstrapping is applied in a variety of different contexts, for instance for a statistical method of resampling (see [2]). Here we use the term bootstrapping in the same way that it is used in the research on judgment and decision making (see [3]). However, we would like to make the reader aware of its different uses in different contexts.

In the judgment and decision-making research on bootstrapping, existing reviews and meta-analyses have suggested that models tend to be more accurate than human judges [4–10]. However, results of previous analyses have also pointed to a wide heterogeneity in the success of bootstrapping [8]. In a previous study [11], we suggested that the success of bootstrapping might depend on the decision domain (e.g., medical or business) as well as on the level of expertise of the decision makers.

To date, however, no meta-analysis has systematically evaluated the success of bootstrapping models across different decision domains or based on the expertise of the human decision maker. Furthermore, to date no review has compared the success of bootstrapping models as a function of the type of evaluation criterion for what constitutes an ‘accurate’ decision. We therefore do not know if bootstrapping is more successful if the evaluation criterion is, for instance, objective, subjective, or a test score (e.g., a student’s test score versus a teacher’s judgment of student performance). Finally, as previous meta-analyses did not correct for measurement error or other methodological artifacts [9], the extent of possible bias in the results of these analyses is currently unknown.

In this study, we conduct a meta-analysis of the success of bootstrapping using the lens model framework. We investigate whether the success of bootstrapping varies across decision domains (e.g., medical or business), the expertise of the human decision maker (expert or novice), or the criterion for a ‘successful decision’ (objective, subjective, or based on a test score). We then compare the results of traditional, ‘bare-bones’ meta-analysis (i.e., only corrected for sampling error, see [12] p. 94) with the results of psychometric meta-analysis in which we were able to correct for a number of potential methodological artifacts [12]. It should be noted that we applied psychometric corrections in a previous paper [11] and that we are using these psychometric-corrected indices for a more comprehensive evaluation of bootstrapping models in the present paper. Hence, the part on the psychometric analysis in our previous study is closely linked to the work presented here, as we used the results of a previous analysis for additional evaluations presented in this paper in the following. We would like to make the interested reader aware that the scope of our previous work was different than in the following. In addition to that, the criteria for including studies in the two meta-analyses are different (e.g., our first paper focused on the evaluation of single lens model indexes, whereas our present paper focuses on a combination of lens model indexes). This study covers issues not considered in our first paper. For example, we also consider expertise level within domains and evaluation criteria. Hence, this paper is an extension of the first one, which supplements it. The link between the two papers is the second database in this paper (see ‘study identification’ and ‘second database’ below), which we reused from our first paper. Hence, also our analytical strategy applying to the second sample depends on our previous analysis, which was presented in our first paper.

Taken together, by adding an additional database, an alternative analytical strategy, and a comparison of the results, we scrutinize the validity of our conclusion that bootstrapping is actually successful. Due to the additional check with this second paper, we also gain greater and more detailed insights into the evaluation of the success of bootstrapping models.

Importantly, the studies in both papers represent exclusively decision-making tasks that mirror actual, real-life decision-making conditions most closely, thus providing the most appropriate evaluation of bootstrapping [13].

### Success of bootstrapping: Previous research

The success of bootstrapping models has been evaluated in several reviews, beginning with Meehl’s seminal evaluation in his book, *Clinical Versus Statistical Prediction* [14]. In this first systematic review of the success of bootstrapping, Meehl summarized 20 studies and concluded that models led to better decisions than decisions made by humans, jumpstarting the “man versus model of man” debate. Since then, several meta-analyses have evaluated the success of bootstrapping, following either a traditional or a lens model approach, as outlined below.

#### Traditional approaches

Reviews taking a traditional approach have generally concluded that models lead to more accurate decisions than human judgment does, although the results have also pointed to heterogeneity in the success of bootstrapping. For instance, based on the results of a meta-analysis of 136 studies, Grove et al. [7] concluded that model prediction was typically as accurate as or more accurate than human prediction, but they noted that there were also some instances in which human prediction was as good as or even better than model prediction. Notably, the results of Grove et al. [7] were specific to medical and psychological decisions and do not necessarily generalize to other decision domains (e.g., nonhuman outcomes such as horse races, weather, or stock market prices). Tetlock [10] and Aegisdottir et al. [4] reached similar conclusions based on their respective reviews of political predictions and counseling tasks. Finally, focusing on potential domain differences in the success of bootstrap models across psychological, educational, financial, marketing, and personnel decision-making tasks, Armstrong [5] concluded that bootstrapping led to more accurate decisions in eight tasks, less accurate decisions in one task, and equally accurate decisions in two tasks.

#### Lens model approach

Relative to other approaches, one advantage of using the lens model framework to evaluate the success of bootstrapping is that one can take into account different human judgment and decision-making strategies. Different kinds of models can be used to bootstrap decision processes. Ecological or actual models are based on the past observed relationship between any number of pieces of information (cues) and a particular outcome. An example of an ecological model is when a linear multiple regression equation based on the past observed relationship between a number of cues (e.g., breast tumor, family history) and actual breast cancer disease is used to make a breast cancer diagnosis [9]. Whereas ecological models ignore human judgment and decision-making strategies, bootstrapping models in the lens model approach take into account the different ways in which decision makers integrate different pieces of information to reach a decision (i.e., non-linear vs. linear). With a non-linear decision-making strategy, the decision maker (e.g., physician) uses a single piece of information, such as whether or not a breast tumor is present. The fast-and-frugal heuristic is a well-known non-linear model (e.g., [15]). Although such non-linear models are generally considered to be particularly user friendly (see e.g., [16]), research has predominantly focused on linear bootstrap models that include multiple cues. Hence, in addition to the presence or absence of a breast tumor, a physician might also consider additional information, such as whether there is a family history of breast cancer. Taking into account such a linear decision-making strategy is also possible within the lens model framework [17, 18]. Thus, using the lens model framework to analyze the success of linear bootstrap models offers the best way to evaluate the success of bootstrapping.

#### The success of bootstrapping by lens model indices

Within the lens model framework, the success or ‘judgment achievement’ of a decision-making process is expressed by the lens model equation, which is a precise, mathematical identity that describes judgment achievement (*r*_*a*_) as the product of knowledge (*G*), environmental validity (*R*_*e*_), and consistency (*R*_*s*_) plus an unmodeled component (*C*) (see Eq 1):

$$r_{a}=G\;R_{s}R_{e}+C\sqrt{1-R_{s}^{2}}\sqrt{1-R_{e}^{2}}$$ (1)

where

*r*_*a*_ = the achievement index (i.e., the correlation between a person’s judgment and a specific criterion),

*R*_*e*_ = the environmental validity index (i.e., the multiple correlation of the cues with the criterion),

*R*_*s*_ = consistency (i.e., the multiple correlation of the cues with the person’s estimates),

*G* = a knowledge index, which is error-free achievement (i.e., the correlation between the predicted levels of the criterion and the predicted judgments), and

*C* = an unmodeled knowledge component, which is the correlation between the variance not captured by the environmental predictability component or the consistency component (i.e., the correlation between the residuals from the above achievement index).

According to Camerer [6] and Goldberg [19], the product of the components knowledge (*G*) and environmental validity (*R*_*e*_) captures the validity of the bootstrapping model. By including the knowledge component (*G*) in the evaluation of the bootstrapping model, we assume that the human judge uses a linear judgment and decision-making strategy, that is, that the judge integrates at least two pieces of information. The degree to which replacing a human judge with a decision-making model improves the success of the decision-making process can be quantified by subtracting judgment achievement from the product term *GR*_*e*_ (see [6] p. 413, see Eq 2).

$$\Delta =GR_{e}-r_{a}$$ (2)

Reviews using the lens model framework and the lens model equation have included ecological models (see [9]) as well as models considering the judgment and decision-making strategy (i.e., linear vs. non-linear). The classic review by Camerer [6] on the success of linear bootstrap models supported the conclusion that bootstrapping with linear models works well across different types of judgment tasks. However, it should be noted that Camerer [6] included laboratory tasks in his review, in violation of the demand for ecological validity applying to studies in the lens model tradition. The results of the more recent analysis by Karelaia and Hogarth [8] were in line with Camerer [6], although the authors pointed out the high heterogeneity of the success of bootstrapping across tasks and highlighted the need to identify the task and judge characteristics that favor bootstrapping. Previous reviews on lens model indices indicated wide heterogeneity (see [20]) and implied domain differences in lens model statistics (see [21, 11]), suggesting that judgment achievement is different in different decision domains (e.g., medicine, business, education, psychology) and in turn implying that the success of bootstrapping models is also domain-dependent. Indeed, these preliminary results suggesting that the success of bootstrapping was domain-dependent highlight the need for more detailed analysis. Hence, this paper extends our previous paper (see [11]).

### The present study

In this study, we conduct a meta-analysis of lens model studies to evaluate the success of linear bootstrapping models. Our meta-analysis is unique and extends our previous paper by focusing specifically on differences in the success of bootstrapping based on the decision domain, the expertise of the human decision maker (expert or novice), and the criterion for an accurate decision (objective, subjective, or test score). An analysis of this kind is needed to identify specific contexts in which bootstrapping is likely to be more successful. In addition, in a second evaluation, we use psychometrically corrected lens model values to construct the bootstrapping model. Previous reviews have not corrected for potential artifacts (e.g., measurement error), which potentially leads to biased estimations [9]. We are therefore the first to evaluate the success of psychometrically-corrected bootstrapping models in detail.

## Methods

Before describing our study identification strategy and databases in detail, we describe the two different analytical strategies used in this study. As different conditions are required for each analytical strategy, we had two different databases. Hence, we report the process of study identification and give detailed study descriptions for the two databases separately.

### Study identification

#### First database

**Fig 1:**
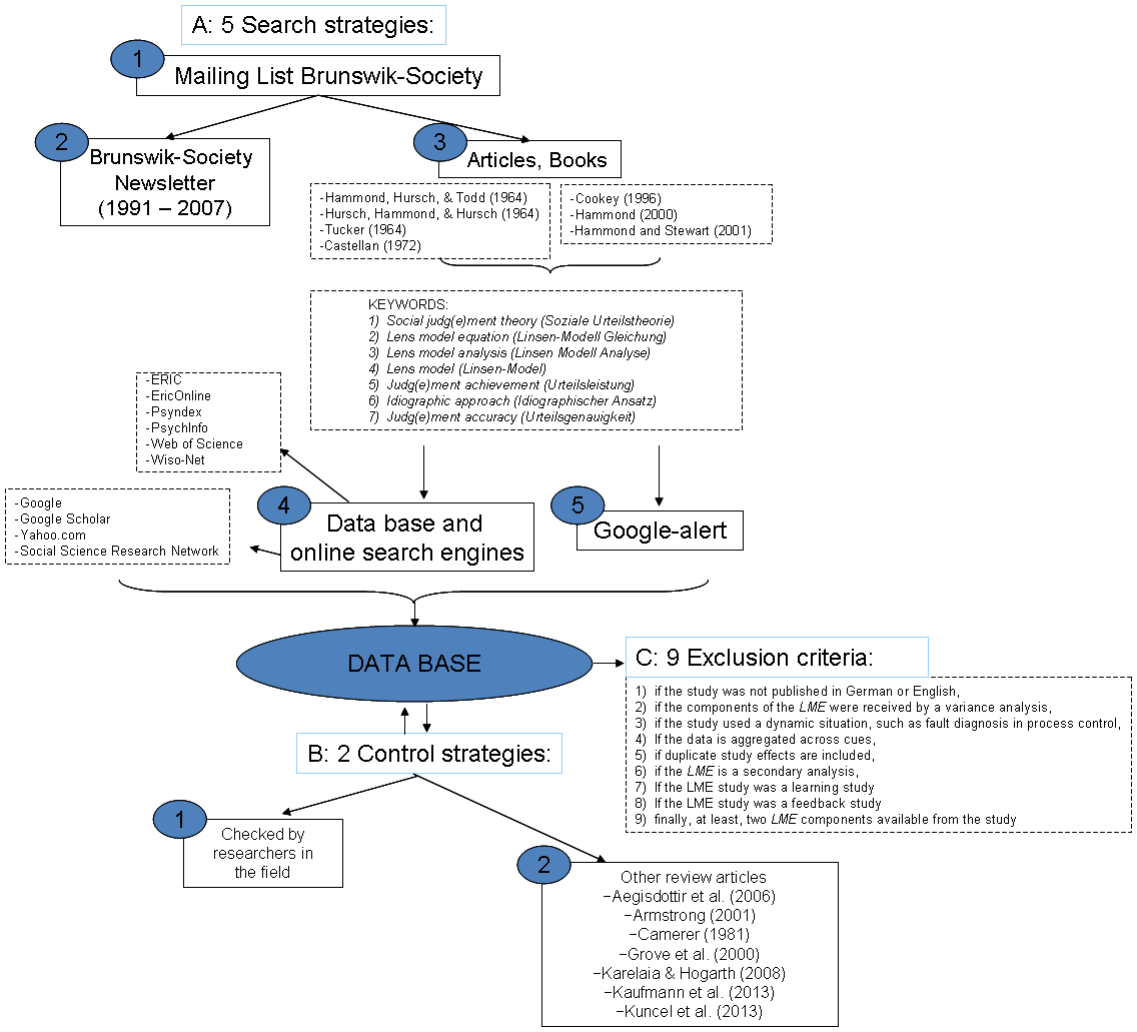
The process of identifying relevant studies for the meta-analysis.

To identify lens model studies to be included in the meta-analysis, we checked the database by Kaufmann et al. [11] as well as the studies included in Camerer [6] and Kuncel et al. [9] (Fig 1). Please note that Kaufmann et al. [11] focused on artifact correction of the lens model components as opposed to the success of bootstrapping models as in this study; hence, they excluded some of the studies included in Camerer [6] from their database. We excluded all studies with feedback or learning opportunities (e.g., [22]; for details we refer to [11]). We argue that excluding studies in which decision makers received feedback on the accuracy of their decisions is more appropriate for evaluating the success of human judgment accuracy relative to bootstrapping in real-life conditions, in which human decision makers rarely receive such feedback.

Tables 1 and 2 show the lens model studies identified through the search procedure, organized by decision domain and decision-maker expertise (expert versus novice). In sum, 35 studies met the inclusion criteria for the first meta-analysis.

**Table 1:** Studies included in the meta-analyses by decision domain and decision-maker expertise.

Study		Judges	Number of judgments	Number of cues	Judgment task	Criterion	Task results
*a)*	*Medical science*, *experts*:						
1)	Nystedt & Magnusson [23]	4 clinical psychologists	38	3	Judge patients based on patient	Rating on three	*I*: *Δ*_*1*_ = .11
					protocols:	psychological tests (■)	*II*: *Δ*_*2*_ = .03
					*I*: intelligence		*II*: *Δ*_*3*_ = .12
					*II*: ability to establish contact		(*, +, s)
					*III*: control of affect and impulses		
2)	Levi [24]	9 nuclear medicine	280	5	Assess probability of significant	Coronary angiography	*Δ*_*4*_ = .07
		physicians	(60 replications)		coronary artery disease based on patient		(*, s)
					profiles		
3)	LaDuca, Engel, & Chovan [25]	13 physicians	30	5	Judge the degree of severity	A single physician’s	*Δ*_*5*_ = .08
					(congestive heart failure) based on	judgment (▲)	(*, s)
					patient profiles		
4)	Smith, Gilhooly, & Walker [26]	40 general practitioners	20	8	Decision to prescribe an antidepressant	Guideline expert (▲)	*Δ*_*6*_ = -.05
					based on patient profile		(s)
5a)	Einhorn [27] (This publication	3 pathologists	*III*: 193	9	Evaluate the severity of Hodgkin’s	Actual number of	*III*: *Δ*_*7*_ = -.01
	contains two studies)				disease based on biopsy slides	months of survival	(s)
	*Second study*						
6a)	Grebstein [28]	10 clinical experts	30 profiles	10	Judge Wechsler-Bellevue IQ scores	IQ test scores (■)	*Δ*_*8*_ = -.17
		(varying in amounts of			from Rorschach psychograms		*Δ*_*9*_ = -.14
		clinical experience)					
5b	Einhorn [27]	29 clinicians	I: 77 MMPI profiles	11	Judge the degree of neuroticism-	Actual diagnosis (■)	*Δ*_*10*_ = .02
	First study (This publication		II: 181 MMPI profiles		psychoticism		*Δ*_*110*_ = -.05
	Contains two studies)						(*, +, s)
7)	Todd (1955, see [29]), Note 3	10 clinical judges	78	19	Estimate patient IQ from the Rorschach	IQ test scores (■)	*Δ*_*12*_ = .05
					test		
8)	Speroff, Connors, & Dawson	123 physicians:	440	32	Judge intensive care unit patients’	Patients’ actual	*Δ*_*13*_ = .05
	[30]	105 house staff,			hemodynamic status	hemodynamic status	(s)
		15 fellows,			(physicians’ estimation)		
		3 attending physicians					
	*Novices*:						
6b)	Grebstein [28]	5 students	30	10	Judge Wechsler-Bellevue IQ scores	IQ test scores (■)	*Δ*_*14*_ = -.19
					from Rorschach psychograms based on		
					paper profiles		
*b)*	*Business science*, *experts*:						
9)	Ashton [31]	13 executives, managers,	42	5	Predict advertising sales for *Time*	Actual advertising pages	*Δ*_*15*_ = .07
		sales personnel			magazine based on case descriptions	sold	(*, +, s)
10)	Roose & Doherty [32]	16 agency managers	200 / 160	64 / 5	Predict the success of life insurance	One-year criterion for	*Δ*_*16*_ = -.08
					salesmen based on paper profiles	success	(*, +, s)
11)	Goldberg [33]	43 bank loan officers	60	5	Predict bankruptcy experience based on	Actual bankruptcy	*Δ*_*17*_ = .03
					large corporation profiles	experience	
12)	Kim, Chung, & Paradice [34]	3 experienced loan	119	7	Judge whether a firm would be able to	Actual financial data	*I*: *Δ*_*18*_ = .09
		officers	*I*: 60 big firms,		repay the loan requested based on		*II*: *Δ*_*19*_ = .02
			*II*: 59 small firms		financial profiles		(*, +, s)
13)	Mear & Firth [35]	38 professional security	30	10	Predict security returns based on	Actual security returns	*Δ*_*20*_ = .03
		analysts			financial profiles		(s)
14)	Ebert & Kruse [36]	5 securities analysts	35	22	Estimate future returns of common	Actual returns	*Δ*_*21*_ = .06
					stocks		
15)	Wright [37]	47 students	50	4	Predict price changes for stocks from	Actual stock prices	*Δ*_*22*_ = .06
					1970 until 1971 based on paper profiles		(*, +, s)
					of securities		
16)	Harvey & Harries [38]	24 psychology students	40	Not	Forecast sales outcomes based on paper	Actual sales outcome	Δ_23_ = -.07
	(1. experiment)			known	profiles		(s)
17)	Singh, 1990 [39]	52 business students	35	Not	Estimate of the stock price of a	Actual stock prices	*Δ*_*24*_ = .02
				known	company based on paper profiles		(s)
*c)*	*Educational science*, *experts*:						
18)	Dawes [40]	1 admission committee	111	4	Admission decision for graduate school	Faculty ratings of l	*Δ*_*25*_ = .06
					based on paper profiles	performance in graduate	
						school (▲)	
19)	Cooksey, Freebody, & Davidson	20 teachers	118	5	Judge I: Reading comprehension	*I-II*: End-of-year test	*I*: *Δ*_*26*_ = .04
	[41]				And II: Word knowledge of	scores (■)	*II*: *Δ*_*27*_ = .04
					kindergarten children based on paper		(*, +, s)
					profiles		
	*Novices*:						
20)	Wiggins & Kohen [42]	98 psychology graduate	110	10	Forecast first-year-graduate grade point	Actual first-year-	*Δ*_*28*_ = .17
		students			averages based on paper profiles	graduate grade point	(s)
						averages	
21)	Wiggins, Gregory, & Diller,	41 psychology students	90	10	Forecast first-year-graduate grade point	Actual first-year-	*Δ*_*29*_ = .06
	see Dawes and Corrigan [43],				averages based on paper profiles	graduate grade point	
	repl. Wiggins and Kohen [42]					averages	
22)	Athanasou & Cooksey [44]	18 technical and further	120	20	Judge whether students are interested in	Actual level of students’	*Δ*_*30*_ = .07
		education students			learning based on paper profile	interest	(*, +, s)
*d)*	*Psychological science*, *experts*:						
23)	Szucko & Kleinmuntz [45]	6 experienced polygraph	30	3–4	Judge truthful / untruthful response	Actual theft	*Δ*_*31*_ = -.06
		interpreters			based on polygraph protocols		(*, +, s)
24)	Cooper & Werner [46]	18	33	17	Forecast violent behavior during the	Actual violent behavior	*Δ*_*32*_ = .00
		(9 psychologists,			first six months of incarceration based	during the first six	(s)
		9 case managers)			on inmates’ data forms	months of imprisonment	
25)	Werner, Rose, Murdach, &	5 social workers	40	19	Predict imminent violence of	Actual violent acts	*Δ*_*33*_ = .03
	Yesavage [47]				psychiatric inpatients in the first 7 days	in the first 7 days	(*, +, s)
					following admission based on	following admission	
					admission data		
26)	Werner, Rose, & Yesavage [48]	30	40	19	Predict male patients’ violent behavior	Actual violence during	*Δ*_*34*_ = .06
		(15 psychologists,			during the first 7 days following	the first 7 days following	(s)
		15 psychiatrists)			admission based on case material	admission	
	*Novices*:						
27)	Gorman, Clover, & Doherty [49]	8 students	75:	*I*, *III*: 12	Predict students’ scores on an attitude	Actual data:	*I*: *Δ*_*35*_ = .73
			*I*, *III*: 50	*II*, *IV*: 6	scale (*I*, *II*) and a psychology	*I*, *II*: Attitude scale	*II*: *Δ*_*36*_ = .67
			*II*, *IV*: 25		examination (*III*, *IV*) based on	*III*, *IV*: Examination scale	*III*: *Δ*_*37*_ = .01
					interviews (I, III) and paper profiles	(■)	*IV*: *Δ*_*38*_ = .29
					(II, IV)		(*, s) (.08), see
							Camerer [6]
28)	Lehman [50]	14 students	40	19	Assess imminent violence of male	Actual violent acts in the	*Δ*_*39*_ = -.01
					patients in the first 7 days following	first 7 days following	(*, +, s)
					admission based on case material	admission	

▲ = subjective criterion;

■ = test criterion;

(*) = idiographic approach (cumulating across individuals);

(*, +) = both research approaches are considered;

Δ = the success of bootstrapping models (see Eq 2); s = sub-sample of tasks for the second evaluation (psychometric corrected bootstrapping models).

**Table 2:** Miscellaneous studies included in the meta-analysis.

Study		Judges	Number of judgments	Number of cues	Judgment task	Criterion	Domain	Task results
*e)*	*Miscellaneous domains*, *experts*:							
29)	Stewart [51]	7 meteorologists	75 (25)	6	Assess probability of	Observed event	Meteorology	*Δ*_40_ = -.01
					hail or severe hail based on radar volume			(*, s)
					scans			
	*Both experts and novices*:							
30)	Stewart, Roebber, & Bosart [52]	4	*I*: 169	12	Forecast 24-h maximum temperature,	*I*, *II*: Actual	Meteorology	*I*: *Δ*_41_ = .00
		(2 students,	*II*: 178	13	12-h minimum temperature,	temperature		*II*: *Δ*_42_ = .00
		2 experts)	*III*: 149	24	12-h precipitation, and	*III*, *IV*: Actual		*III*: *Δ*_43_ = .00
			*IV*: 150	24	24-h precipitation for each day	precipitation		*IV*: *Δ*_44_ = .00
								(*, +, s)
	*Novices*:							
31)	Steinmann & Doherty [53]	22 students	192:	2	Decide which of two randomly chosen	A hypothetical	Other	*Δ*_45_ = .15
			(2 sessions with 96		bags a sequence of chips had been drawn	“judge”		(*, s)
			judgments)			(▲)		
32)	MacGregor & Slovic [54]	*I*: 25 students	*I—IV*:	4	Estimate the time to complete a marathon	Actual time to	Sport	*I*: *Δ*_46_ = .19
		*II*: 25 students	40		based on runner profiles	complete the		*II*: *Δ*_47_ = .16
		*III*: 26 students				marathon		*III*: *Δ*_48_ = .23
		*IV*: 27 students						*VI*: *Δ*_49_ = .24
								(s)
33)	McClellan, Bernstein, & Garbin	26 psychology	128	5	Estimate magnitude of fins-in and fins-out	Actual magnitude	Perception	*Δ*_50_ = .12
	[55]	students			Mueller Lyer stimuli	of fins-in and fins-		(s)
						out Mueller Lyer		
						stimuli		
34)	Trailer & Morgan [56]	75 students	50	11	Predict the motion of objects based on	Actual motion	Intuitive	*Δ*_51_ = .10
					situations in a questionnaire		physics	(*, +, s)
35)	Camerer [57]	21	18	—	—	—	—	*Δ*_52_ = .00

▲ = subjective criterion;

(*) = idiographic approach (cumulating across individuals);

(*, +) = both research approaches are considered;

Δ = the success of bootstrapping models (see Eq 2); *s* = subsample of tasks for the second evaluation (psychometric corrected bootstrapping models).

#### Second database

A subset of 31 studies in the database described above met the inclusion criteria for the evaluation of artifact-corrected bootstrapping models (see [11]). In Tables 1 and 2, this subset of studies is labeled with an ‘s’ for subsample in the last column. We also point out here that in contrast to the first database, the second database is the same as in Kaufmann et al. [11]

Further details on the construction of our databases, such as our search protocol, are available in Kaufmann [58].

### Study descriptions

#### First database

We identified studies within five decision domains: medical science (8 studies), business science (9 studies), educational science (5 studies), psychological science (6 studies), and miscellaneous (7 studies). Most judgments were based on paper profiles, i.e., written descriptions (see [59]). Overall, the number of cues ranged from two [53] to 64 [32]. The number of decision makers in the studies ranged from three [27, 34] to 123 [30]. The majority of the studies included novice decision makers (predominantly students). The number of decisions ranged from 25 [26] to 440 [30]. The meta-analysis included evaluation of 52 different decision tasks. Tables 1 and 2 also describe the criterion in each study. Notably, some studies included an objective criterion, such as the actual weather temperature (see [52]), and other studies included a subjective criterion, such as a physician’s judgment (see [25]). Subjective criteria are indicated by black triangles in Tables 1 and 2, and test score criteria (e.g., [23]) are indicated by a square. Criteria not specially labeled are objective criteria.

As Table 1 shows, we identified eight studies within medical science, which included 241 experts (e.g., clinical psychologists) and five novices and 14 different tasks. The studies within the medical science domain included the studies with both the overall lowest and the overall highest number of judgments. In the first study by Einhorn [27], the three pathologists were the only decision makers who based their judgments on real biopsy slides, which represented a more natural situation than the commonly used paper patient profiles. We identified nine studies within business science, including 40 bootstrapping models by 241 persons for 10 different tasks. Please note that the study by Wright [37] analyzed only the five most accurate judgments made by the 47 persons included at the idiographic level. Studies within business science had the widest range of number of cues (4 to 64). All judgments were based on paper profiles. We identified five studies within educational science, two studies with expert decision makers and three with novice decision makers. In the two studies with experts, 41 bootstrapping models in three tasks were considered. Cooksey, Freedbody, and Davidson [41] included a multivariate lens model design, supplemented with two single lens model designs. In the present analysis, we used the two single lens model designs as two different tasks. We identified six studies within psychological science, in which 105 bootstrapping models of 81 individuals (including 59 experts) for nine different tasks were available. Finally, we identified seven studies that did not fit into any of the other domain categories (e.g., studies on the accuracy of weather forecasts). The studies in the miscellaneous domain included data from 258 individuals (9 experts vs. 249 novices) for 13 different tasks and 270 bootstrapping models. Please note that only Stewart, Roebber, and Bosart [52] directly compared novices and experts across four meteorological tasks. It is also the only study within the miscellaneous domain to have analyzed judgment accuracy retrospectively.

In sum, in our meta-analysis we analyzed the results of 35 studies with 1,110 bootstrapping models, 532 experts, and 578 novices judging 52 tasks across five decision domains. This sample also includes 365 bootstrapping procedures at the individual level (idiographic approach) across 28 different tasks.

#### Second database

The subset of 31 studies (the second database) with sufficient information for evaluating the success of bootstrapping with psychometrically-corrected lens model indices included 1,007 bootstrapping models, covering 44 tasks across five decision domains (see [11], for more information).

### Analytic strategy

Based on our preliminary analysis of the success of individual bootstrapping procedures at the individual level, we now outline our two analytical strategies. Please keep in mind that in each of these analytical strategies, a different sample was included, as described above. Moreover, in line with previous work (see [8, 11]) the analytical level was that of tasks, not studies. The included effect sizes for the success of the model for each task in our meta-analysis can be found in the last column in Table 1.

#### The success of individual bootstrapping procedures

In meta-analysis, an ecological fallacy may arise because associations between two variables at the group level (or ecological level) may differ from associations between analogous variables measured at the individual level (see [60]). For this reason, we plotted the success of individual bootstrapping procedures first before analyzing the aggregated estimation of success of bootstrapping calculated through meta-analysis (see the next step in the analysis).

#### Bare-bones meta-analysis

We used the lens model equation to calculate the success of bootstrapping (see final results column of Table 1 for the indices of the success of bootstrapping models). Our bare-bones meta-analysis strategy was in line with the analysis approach used by Karelaia and Hogarth [8] in their meta-analysis. Moreover, in line with the review by Camerer [6] and Karelaia and Hogarth [8], we included the linear knowledge component in our estimation of bootstrapping success. Hence, we underestimated general success, as the knowledge component was smaller than 1, leading to a decrease of the model component in contrast to Kuncel et al. [9], who excluded the knowledge component (*G*) from their evaluation of bootstrapping success. Thus, we gained more information about the human judgment and decision-making strategy than was possible in Kuncel et al. [9].

We followed the Hunter-Schmidt approach to meta-analysis [12]. The Hunter-Schmidt approach estimates the population effect size by correcting the observed effect size for bias due to various artifacts, including sampling and measurement error (see [12], p. 41). Specifically, we corrected for possible sampling bias introduced by the different number of judges in the single studies, using what is referred to as bare-bones meta-analysis. We used forest plots to graphically analyze the results of the bare-bones meta-analysis. We were specifically interested in whether the success of bootstrapping depended on decision domain, the level of expertise of the human judge, or the type of criterion. Hence, for this moderator analysis, we reran the meta-analysis with a subsample of studies.

In addition to the overall success of models (see the third column in Tables 3 and 4), we also report the confidence and the credibility intervals (see fourth and fifth columns of Tables 3 and 4). In contrast to confidence intervals, credibility intervals are calculated with standard deviations after removing artifacts and correction of sample bias. If the credibility interval includes zero or is sufficiently large, there is a higher potential for moderator variables relative to when the credibility interval is small and excludes zero (for further information, see [61]). We considered additional estimations of heterogeneity to the Q-test: If this test is significant, moderator variables are indicated (see sixth and seventh columns of Tables 3 and 4). The I^2^ ([62], see eighth column of Tables 3 and 4) represents the between-task heterogeneity not explained by the sampling error; values above 25% indicate variation. Moreover, the *τ*^*2*^ is an additional index for the between-heterogeneity (see the second to last column of Table 3): If *τ*^*2*^ is zero, this implies homogeneity. Finally, we used the 75% rule as an indication of moderator variables (see the last column of Tables 3 and 4). That is, moderators were expected whenever artifacts explained less than 75% of the observed variability.

**Table 3:** Results of the bare-bones meta-analysis organized by decision domain and decision maker’s expertise.

Domains (expertise)	*k*	*N*	*Δ*	*SD*_*Δ*_	*95% CI*	*80% CI*	*Q*	*I*^*2*^*(%)*	*τ*^*2*^	*75%*
Medical	14	293	.00	.00	-.10 - .12	.00 - .00	1.3 ^n.s.^	0.00	0.00	1,171
*Publ*. *bias*	+3	324	.03	.00	-.02 - .04	.03 - .03	39.15**	59.1	0.00	667
Expert	13	288	.01	.00	-.10 - .12	.01 - .01	1.19 ^n.s.^	0.00	0.00	1,262
*Publ*. *bias*	+2	305	.02	.00	-.02 - .04	.02 - .03	36.59***	61.7	0.00	895
Novice	—	—	—	—	—	—	—	—	—	—
Business	10	244	.02	.00	-.10 - .14	.02 - .02	.49 ^n.s.^	0.00	0.00	2,338
Expert	7	121	.02	.00	-.15 - .20	.02 - .02	.22^n.s.^	0.00	0.00	3,791
Novice	3	123	.00	.00	-.15 - .19	.02 - .02	.26 ^n.s.^	0.00	0.00	1,146
*Publ*. *bias*	+1	125	.02	.00	-.01 - .09	.02 - .02	15.38***	80.5	0.001	1,686
Education	6	198	.11	.00	-.02 - .25	.11 - .11	.68	0.00	0.00	> 10,000
*Publ*. *bias*	+3	208	.12	.00	.11 - .21	.12 - .12	67.14***	88.1	0.003	> 10,000
Expert	3	41	.04	.00	-.26 - .34	.00 - .00	.00 ^n.s.^	0.00	0.00	> 10,000
Novice	3	157	.13	.00	-.03 - .28	.13 - .13	.42 ^n.s.^	0.00	0.00	707
*Publ*. *bias*	+2	162	.13	.00	.11 - .22	.13 - .13	47.16***	91.5	0.003	1,214
Psychology	9	105	.14	.00	-.05-.33	.14-.14	6.5 ^n.s.^	0.00	0.00	> 10,000
Expert	4	59	.03	.00	-.22 - .28	.03 - .03	.01 ^n.s.^	0.00	0.00	4,971
*Publ*. *bias*	+2	62	.03	.00	.01 - .10	.03-.03	3.31 ^n.s.^	0.00	0.00	> 10,000
Novice	5	46	.29	.00	.00 - .58	.29 - .29	4.59 ^n.s.^	0.00	0.00	102
*Publ*. *bias*	+1	47	.30	.00	-.08 - .49	.3 - .3	67.15***	92.6	0.11	> 10,000
Miscellaneous	13	270	.13	.00	.01 - .25	.13 - .13	1.54 ^n.s.^	0.00	0.00	929
Expert	5	15	.00	.00	-.51 - .50	.00 - .00	.00 ^n.s.^	0.00	0.00	> 10,000
*Publ*. *bias*	+3	27	-.01	.00	-.23 - .21	-.01 -.01	.00 ^n.s.^	0.00	0.00	> 10,000
Novice	12	255	.14	.00	.02 - .26	.14 - .14	1.25 ^n.s.^	0.00	0.00	1,269
Overall Experts	32	532	.03	.00	-.07 - .10	.03 - .03	1.56 ^n.s.^	0.00	0.00	> 10,000
*Publ*. *bias*	+5	820	.04	.00	.01 - .05	.04 - .04	53.33**	32.5	0.006	> 10,000
Overall Novices	20	578	.12	.00	.03 - .20	.12-.12	9.65 ^n.s.^	0.00	0.00	> 10,000
Overall	52	1,110	.07	.00	.01 - .13	.07 - .07	14.21^n.s.^	0.00	0.00	> 10,000
*Publ*. *bias*	+ 12	1,365	.10	.00	.73 - .12	.10 - .10	398***	84.2	0.005	> 10,000

*k* = number of judgment tasks;

*N* = number of success indices;

*Δ* = the success of bootstrapping models (see Eq 2); *SD*_*Δ*_ = standard deviation of true score correlation; *95% CI* = confidence interval; *80% CI* = 80% credibility interval including lower 10% of the true score and the upper 10% of the true score; *75%* = percent variance in observed correlation attributable to all artifacts; *Publ*. *bias* = publication bias corrected estimation by the trim-and-fill method (see [63]);

+ = the number of missing tasks indicated by the trim-and-fill method.

**Table 4:** Results of the bare-bones meta-analysis of the success bootstrapping organized by type of evaluation criterion.

Evaluation criteria	*k*	*N*	*Δ*	*SD*_*Δ*_	*95% CI*	*80% CI*	*Q*	*I*^*2*^*(%)*	*τ*^*2*^	*75%*
Subjective	4	76	.03	.00	-.19 - .25	.03 - .03	.60 ^n.s.^	0.00	0.00	520
*Publ*. *bias*	+2	81	.02	.00	-.16 - .06	.02 - .02	44.41***	88.7	0.01	> 10,000
Objective	33	857	.08	.00	.01 - .14	.08 - .08	4.78 ^n.s.^	0.00	0.01	778
*Publ*. *bias*	+9	1,020	.10	.00	.06 - .12	.10 - .10	216***	81.1	0.00	639
Test	15	177	.07	.00	-.08 - .21	.07 - .07	8.68^n.s.^	0.00	0.00	197
*Publ*. *bias*	+3	330	-.01	.01	-.12 - .09	-.14 - .11	149.33***	88.6	0.03	86.14

*k* = number of judgment tasks;

*N* = number of success indices;

*Δ* = the success of bootstrapping (see Eq 2);

*SD*_*Δ*_ = standard deviation of true score correlation; *95% CI* = confidence interval; *80% CI* = 80% credibility interval including lower 10% of the true score and the upper 10% of the true score; *75%* = percent variance in observed correlation attributable to all artifacts; *Publ*. *bias* = publication bias-corrected estimation by the trim-and-fill method (see [63]); + = the number of missing tasks indicated by the trim-and-fill method.

As mentioned above, for our moderator analysis we reran the analysis for each decision domain, for experts and for novices, and for the level of expertise in the domain. We also reran the analysis for each type of evaluation criterion (objective, subjective, or test score) separately.

We then checked our results with a sensitivity analysis. First, we checked for possible publication bias using the trim-and-fill method (see [63]). This approach estimates the effect sizes of potentially missing studies and considers them within a new meta-analysis estimation. Second, we used the leave-one-out approach to check whether the results were influenced by any individual task. In this approach, the first task is excluded in an initial meta-analysis. Then in a subsequent analysis, only the second task is excluded. Hence, for example, for our overall meta-analysis with 52 tasks, 52 separate meta-analyses including 51 tasks were conducted and the results were compared.

#### Artifact-corrected lens model indices

To check the robustness of the results of the bare-bones meta-analysis, we used the subset of *k* = 31 tasks with sufficient information to evaluate the success of artifact-corrected bootstrap models using the psychometrically-corrected lens model components from Kaufmann et al. [11]. In the same way, we also used these databases with lens model indices corrected by a bare-bones meta-analysis to check the differences between the two approaches directly. This procedure was also applied in Kaufmann et al. [11]. It should be noted that here, we used meta-analysis-corrected indices, in contrast to the previously described analytical strategy, in which the indices were not corrected before building the bootstrapping models. In our presentation of this second analytical strategy, we consider the five domains and judge expertise.

## Results

### The success of individual bootstrapping procedures

**Fig 2:**
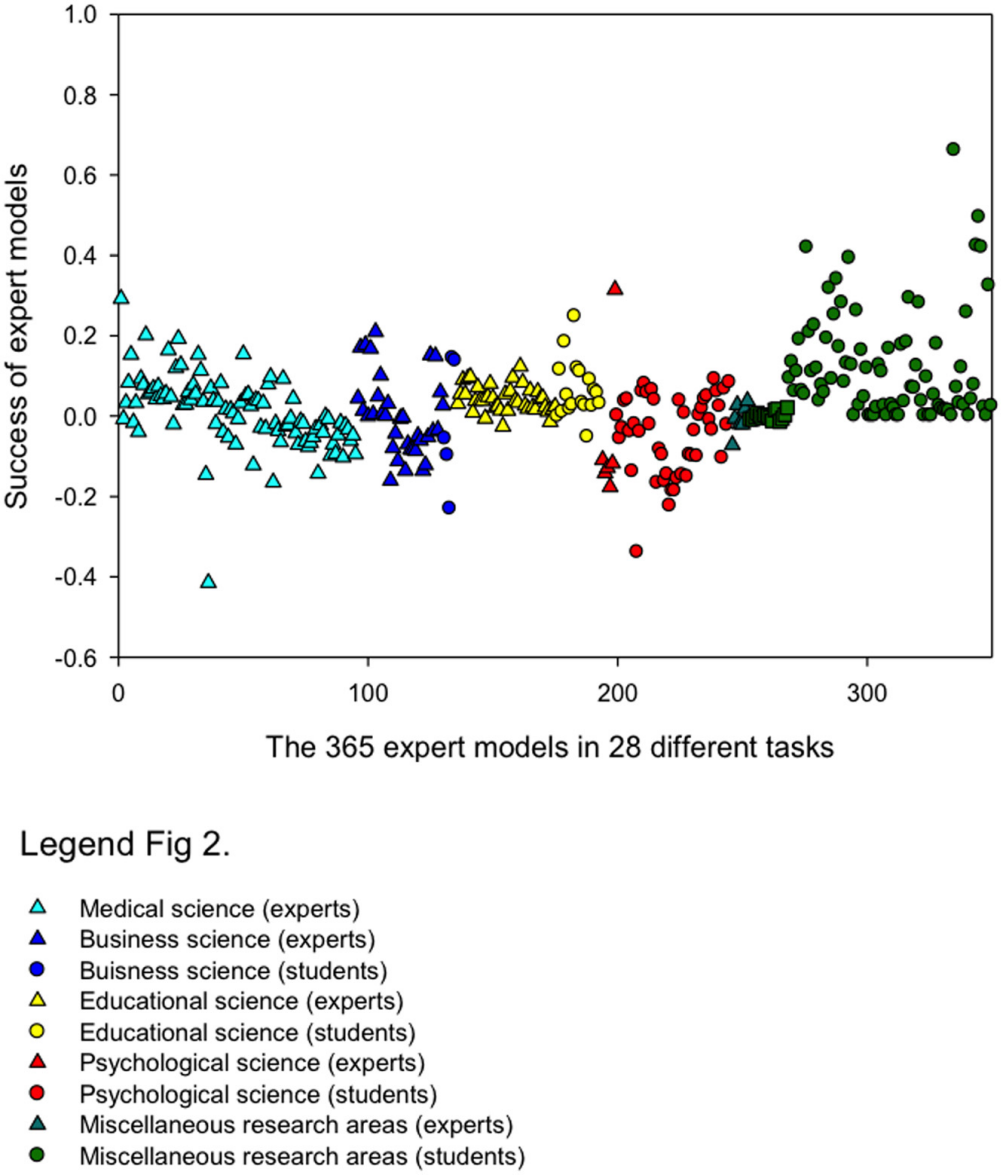
Scatter plot of the success of 365 bootstrapping procedures across 28 different tasks organized by decision domain and decision maker expertise.

Fig 2 displays a scatter plot of the success of 365 individual bootstrapping procedures (see Eq 1), organized by domain (marked by color) and decision maker expertise (triangles for experts, circles for novices). A value of zero indicates that the model was as accurate as the human judge; positive values indicate that the model was more accurate than the human judge. The scatter plot displays the wide variability in the success of the bootstrapping models.

### Bare-bones meta-analytic results

**Fig 3:**
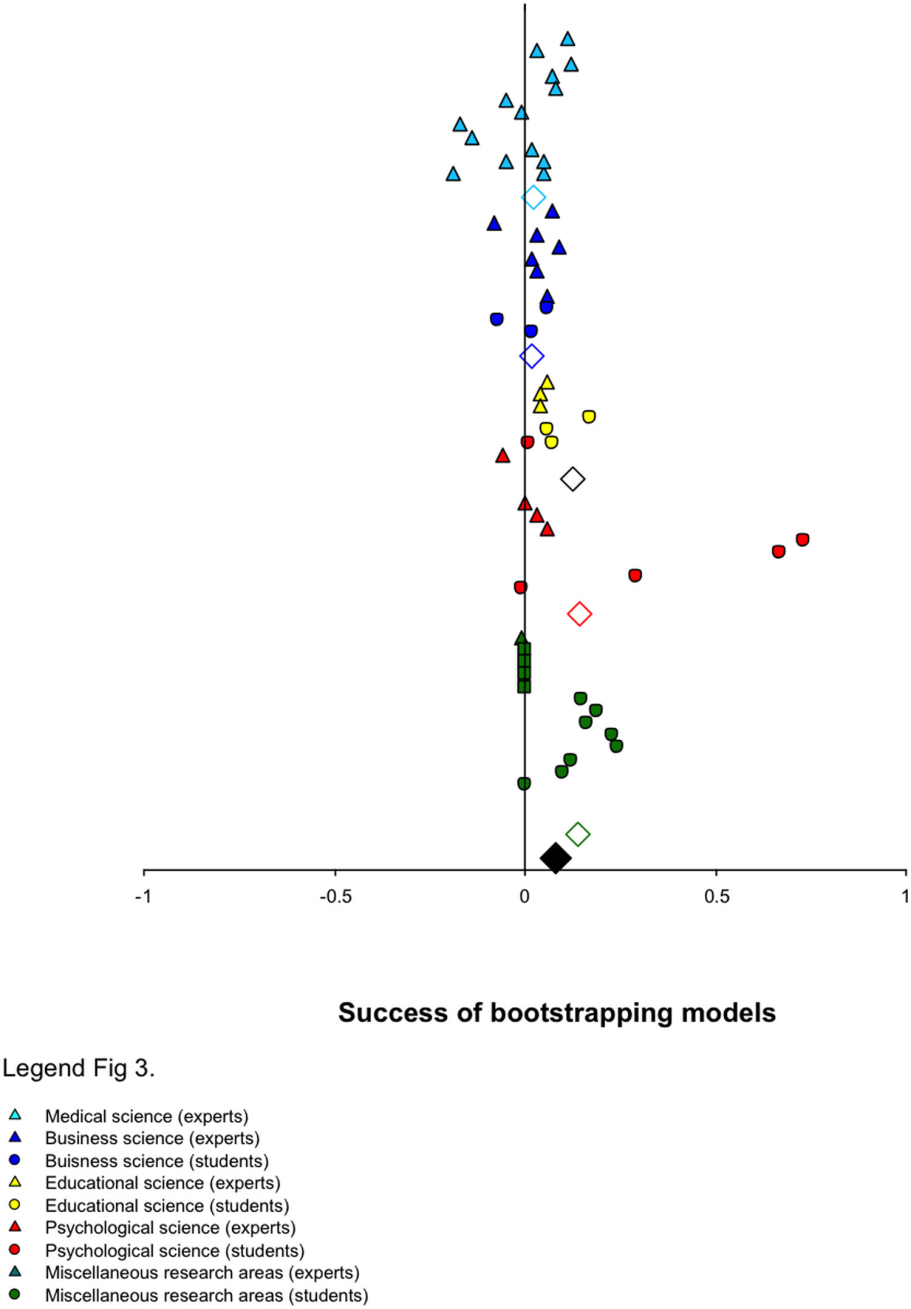
Forest plots of the success of bootstrapping models organized by decision domain and decision maker expertise. Positive values indicate that bootstrapping resulted in more accurate judgments than human judgment.

Fig 3 shows the forest plots. More than 80% of the tasks (42 of the 52 tasks) were associated with a positive value, indicating that the bootstrapping models were more accurate than the human judges. Particularly noteworthy is that bootstrapping was more accurate than human judgment across all of the tasks within education sciences.

Across all tasks, the results of the bare-bones meta-analysis demonstrated that models were generally more accurate than human judges (Δ = .071 across all tasks, see Table 3). There was no indication of moderator variables according to the several heterogeneity indices. In contrast, our publication bias estimation revealed that 12 tasks may have been missed. The resulting publication bias-corrected overall estimation of the success of bootstrapping models indicated the possibility of moderator variables. Although not all heterogeneity indexes confirmed the possibility of moderator variables, we undertook the moderator analysis to check our results.

As Table 3 shows, if we focus on the expertise level, our analysis revealed overall that the success of bootstrapping models was greater within the novice category than within the expert category (.12 vs. .03). Within the different domains, models were generally more successful relative to novice judgment than relative to expert judgment, with the exception of business decisions. Within the business decision domain, models with expert judges were more successful than models with novice judges. There was no indication of moderator variables across the different heterogeneity indices.

As you see, the results were confirmed by our publication bias estimation within the different fields, except in the medical and educational fields, revealing that our results in these areas may be underestimated (see also the associated confidence intervals) and that additional moderator variables may be indicated.

On the other hand, it should be noted that our leave-one-out approach check revealed that within the educational field, there was a decrease in the success of bootstrapping models if the paper by Wiggins et al. (see [43]) was excluded (Δ = .5, -14–24).

If we now assumed some moderator variables within the different fields and focused on the expertise level (expert vs. novice) within the different fields again, possible publication bias seemed to be associated with an increased success of bootstrapping except in the ‘miscellaneous’ field category. Additionally, publication bias-corrected estimation within this miscellaneous field category and within the psychology expert category revealed, contrary to other publication bias-corrected estimations, no additional moderator variables.

To summarize, all the different analyses (with and without publication corrections, the leave-one-out approach) revealed a positive value of the success of bootstrapping models. The only exception was the publication bias-corrected estimation within the miscellaneous category considering experts. However, we highlight here that the positive value of the success of bootstrapping models was not completely confirmed by the 95% confidence intervals but by our 80% credibility interval estimations, which we discuss below.

Additionally to our reported bare-bones meta-analysis, Table 4 displays the results of the bare-bones meta-analysis separated by evaluation criterion (objective, subjective, or test score). As Table 4 shows, bootstrapping was more successful when there was an objective criterion and less successful when a subjective or test score criterion was used at first glance. If we consider the 95% confidence interval, negative success values were revealed within the subjective and the test categories. Our analysis of evaluation criteria indicated no possible moderator variables across the different heterogeneity indices. Additionally, in each evaluation criteria category, a publication bias was indicated by the trim-and-fill approach. Our reanalysis considering a possible publication bias affecting the success of bootstrapping suggested that the success of models was underestimated in the objective evaluation criteria category and overestimated in the subjective evaluation criteria category. Within all evaluation criteria categories, the publication bias-corrected estimations now indicated possible moderator variables.

### Artifact-corrected results

Table 5 displays the results of the success of bootstrap models with psychometrically-corrected lens model indices (*k* = 31). These results suggest that the success of bootstrapping was in fact clearly greater than the results of the bare-bones meta-analysis suggested (.07 vs. .23). If we compared the results with our previously presented bare-bones meta-analysis (see Table 2), our conclusion was confirmed. Importantly, it should be noted that the artifact-corrected results were based on only a subset of the studies included in the bare-bones meta-analysis, as outlined above. Thus, the results of the previous bare-bones meta-analysis and the artifact-corrected results were not directly comparable. Nevertheless, both results partly indicated that models were more successful than human judges across all decision domains. Notably, in comparison with the results of our previously presented bare-bones meta-analysis, the psychometrically-corrected models indicated a different pattern of results on the success of bootstrapping across levels of expertise and decision domains (see Table 4).

**Table 5:** The success of bootstrapping according to bare-bones (in brackets) and psychometrically-corrected lens model indices.

Domains	*k*	*N*	Δoverall^b^	Δexperts	Δnovices
Medical science	10	258	.35 (.01)	.35 (-.01)	.35 (-.01)
Business	9	239	.018^a^ (-.03)	.05^a^ (-.01)	.09^a^ (-.02)
Education	4	156	.21 (.12)	.18 (.15)	.14 (.04)
Psychology	9	105	.08 (.04)	.23^a^ (.15)	.04 (.04)
Miscellaneous	12	249	.26 (.16)	.27^a^ (.16)	.01 (-.02)
Overall	44	1,007	.23 (.07)	.22 (.13)	.17 (.02)

*k* = number of judgment tasks; *N* = number of success indices; *Δ* = estimated success of bootstrapping (see Eq 2).

^a^ = no correction of the R_e_ component, because this component includes only objective criteria.

^b^ = this column is the same as in Kaufmann et al. [11], Table 7, columns 5 and 6.

## Discussion

Like previous reviews [4, 7, 8], we first used a bare-bones meta-analytic procedure [12] to evaluate the success of bootstrapping. Unique to the present study was our additional use of psychometrically-corrected bootstrap models, which are based on a previous meta-analysis (see [11]). These results allowed us to check for various methodological artifacts that may have biased the results of previous meta-analyses. The major finding of this study is that models lead to more accurate judgments than individual human judges make across quite diverse domains (Δ = .07). The results of the present meta-analysis are in line with previous meta-analyses of the overall success of bootstrapping [6, 8]. Notably, there were 10 tasks in which models were not superior to human judges. We argue that the results of meta-analysis of the success of bootstrap models with artifact-corrected lens model indices represent a more accurate estimation of the success of bootstrapping. Comparison of the results of the success of bootstrap models with artifact-corrected lens model indices with the results of the bare-bones meta-analysis in the present study suggests that previous meta-analyses may have underestimated the success of bootstrapping [4, 7, 8, 9]. Although the estimated success of bootstrapping is only slightly higher according to the results of the meta-analysis examining the success of bootstrap models with artifact-corrected lens model indices relative to the bare-bones meta-analysis, the higher (and more accurate) success estimates are meaningful particularly in high-risk decision-making domains like medical science, in which even a small increase in decision accuracy could lead to many saved lives. In sum, our results support the conclusion that formal models to guide and support decisions should be developed especially in decision domains where the cost of inaccurate decisions is high. It should be noted, however, that we used a slightly reduced subset of tasks in the estimation of the success of bootstrap models with artifact-corrected lens model indices (the same database as [11]) as compared to the bare-bones meta-analysis, so that the two estimates of the success of bootstrapping are not directly comparable.

Moreover, we found that there were no systematic differences in the estimated success of bootstrapping depending on the decision domain. However, we highlight that the success of bootstrapping was particularly high in the psychological decision domain. Based on the success of bootstrapping within psychology in the present study, it seems suitable to apply bootstrapping more widely in psychological decision-making tasks in order to overcome the low judgment achievement of psychological experts (see [21, 11]).

The present analyses also considered the potential role of judge expertise in the success of bootstrapping. The results indicate that not only novices but also experts may profit from bootstrapping (see also [10]). The results of the bare-bones meta-analysis suggest that mainly novices profit from bootstrapping, whereas the results of the psychometrically-corrected lens model indices suggest that mainly experts profit from bootstrapping. We note once again that the samples of studies included in the two analyses differed slightly, and hence, the results are not directly comparable. In light of the inconsistent results on the relationship between bootstrapping success and level of judge expertise, we recommend that future studies also consider expertise as a potential moderator of bootstrapping success. We emphasize that only the study by Stewart, Roebber, and Bosart [52] compared novices and experts across the same four meteorological tasks, and we therefore urge researchers to conduct more studies directly comparing novice and expert judges.

Finally, in the present analysis, we considered the type of evaluation criterion as a potential moderator of the success of bootstrapping. Namely, we analyzed the success of bootstrapping separately for studies in which the accuracy of a decision was based on an objective, subjective, or test criterion. We believe that future evaluations of bootstrapping success should likewise consider the type of decision criterion (see also [7] with regards to human and non-human decision domains). In the present study, we found that bootstrapping was especially successful when there was an objective criterion for an accurate decision (e.g., [54]). The higher success of bootstrapping in tasks with an objective criterion is unexpected, since human judges are thought to receive faster and more definite feedback regarding the accuracy of their decisions when there is an objective criterion relative to subjective criterion [64]. The results of our analysis also imply that the results of the meta-analysis by Grove et al. [7] and Aegisdottir et al. [4] may underestimate the success of bootstrapping, since both of those meta-analyses excluded studies with tasks predicting nonhuman outcomes (e.g., weather forecasts). Hence it is primarily with objective criteria that bootstrapping appears, based on the present results, to be particularly successful. Our publication bias-corrected estimation supports our assumption. However, we note that the sample of studies including subjective criteria is quite small, which may limit the generalizability of our results.

Taken together, our review confirms previous meta-analyses in the field and contributes new knowledge on differences in the success of bootstrapping across different decision domains, different levels of expertise of the human judge, and different types of evaluation criteria.

However, a potential point of criticism in our study is that our conclusions are not confirmed by our interpretation of the confidence intervals. We argue that the confidence interval estimations did not consider any sampling bias, which is considered in the credibility intervals estimations, also reported in our work (see Table 4, 5, [12], p. 228). If we focus on the sampling bias-corrected credibility intervals, our results are clearly supported, except in two cases. These two cases are the publication bias-corrected estimation of the success of models in the miscellaneous expert category and the publication bias-corrected estimation in the evaluation criterion category test. Hence, we argue that especially within these two categories, the success of models may be not confirmed. We also emphasize the need for caution in interpreting our publication-corrected estimations, as these estimations are based on a database without any artifact corrections such as measurement error. Hence, the heterogeneity of our databases may be overestimated due to measurement error (see [11]), leading to an overestimation of a possible publication bias.

Moreover, it is important to note that the scope of the present meta-analysis was limited to the success of linear bootstrap models, which represent only one type of formal decision-making model. Our analysis of only linear models may overestimate the potential success of bootstrapping in general (see [9]), since linear models have the problem of overfitting, in contrast to the fast and frugal non-linear models [65]. Non-linear models are also considered to be more user-friendly, which may increase their application in real-life settings [16]. Notably, as an evaluation of the success of artifact-corrected linear models relative to non-linear models has not yet been conducted, it offers an interesting and important avenue for future research. In addition, we see the need to evaluate how the success of bootstrapping may be affected by the number of cues provided in decision-making tasks (i.e., to examine whether bootstrapping is more successful when human judges are provided with more or less information). Further, we feel that future evaluations of bootstrapping success should consider Brunswik’s symmetry concept (see [66]). Judgment achievement increases if both the judgment and the criterion are measured at the same level of aggregation (i.e., if they are ‘symmetrical’). For example, if a physician is asked to judge whether cancer is present and the criterion is whether a cancer tumor is detected, then the judgment is not symmetrical, as cancer can exist without a detectable tumor. In contrast, if a physician is asked to judge whether there is cancer only when a cancer tumor has been detected, then the judgment and the criterion are said to be symmetrical. We did not control for symmetry in the present analysis, which may have led to an underestimation of the lens model components. Future research on whether the symmetry concept moderates the estimated success of bootstrapping would be highly useful in providing a more thorough understanding of the contexts in which models make better judges than humans do, leading to improved judgment accuracy within different domains.
